# Side Effects of COVID-19 Vaccines in Iraqi Kurdistan: A Population-Based Study

**DOI:** 10.7759/cureus.71170

**Published:** 2024-10-09

**Authors:** Ibrahim A Naqid, Ahmed A Mosa, Lilaz S Hito, Dania S Jamil, Israa T Shukur, Dana S Abdulkareem, Nawfal R Hussein

**Affiliations:** 1 College of Medicine, University of Zakho, Zakho, IRQ

**Keywords:** covid-19 pandemic, covid-19 vaccines, infectious disease control, kurdistan region of iraq, side effects

## Abstract

Background and aim

The COVID-19 pandemic has globally impacted all sectors. Early vaccine development was crucial to curb the spread of the virus. However, concerns about vaccine safety and side effects have led to hesitancy. This study aims to examine and compare side effects associated with Pfizer/BioNTech, Oxford/AstraZeneca, and Sinopharm vaccines in Iraqi Kurdistan.

Materials and methods

A population-based study was conducted in the Kurdistan Region of Iraq from September 2022 to April 2023, involving 1,340 participants recruited through face-to-face interviews and online forms. The questionnaire collected demographic data and information on COVID-19 infection and vaccination status.

Results

Among the participants, 52.76% were females, with a mean age of 29.21 years (±13.09 SD). Of these, 67.84% received the Pfizer/BioNTech vaccine, and 60.9% had a prior COVID-19 infection. About 76.94% experienced post-vaccination side effects, lasting an average of 2.8 days (±1.92 SD). Notably, 60% reported no or mild side effects. Common side effects across all vaccines included injection site pain, fever, headache, and fatigue. Side effects were more frequent after the first dose and were highest with Oxford/AstraZeneca, followed by Pfizer/BioNTech and Sinopharm (p = 0.001). Higher rates of side effects were observed in participants aged 36-60, females, married individuals, those with chronic conditions, previously infected individuals, and those who contracted COVID-19 post-vaccination (p = 0.001).

Conclusions

This study reveals that most of the participants experienced either no side effects or only mild reactions following vaccination, with none of the side effects being serious. These findings are expected to boost public confidence and increase vaccine uptake, especially with booster doses now available.

## Introduction

The COVID-19 pandemic spread rapidly across the globe, placing immense strain on healthcare systems worldwide, including those in the Kurdistan Region of Iraq [1,2]. In response to the escalating crisis, governments around the world implemented a range of preventive measures to curb the transmission of the virus [3,4]. Despite these efforts, the virus continued to proliferate, manifesting in diverse clinical presentations across different regions, reflecting global patterns [5,6]. Achieving herd immunity, a crucial goal for controlling and eventually ending the pandemic hinges on widespread and effective vaccination. In a remarkable display of global scientific collaboration, multiple COVID-19 vaccines were developed and approved in a relatively short period [7,8]. The rapid distribution and uptake of these vaccines became a critical focus globally, as countries sought to decrease the infection rates and restore normalcy.

The World Health Organization (WHO) has granted emergency use authorization to several COVID-19 vaccines. However, only three vaccines were approved and administered in the Kurdistan Region of Iraq including Pfizer/BioNTech mRNA-based vaccine (BNT162b2), Oxford/AstraZeneca vaccine (ChAdOx1 nCoV-19), and Sinopharm (BBIBP-CorV) [9]. Unfortunately, numerous rumors and misconceptions regarding various aspects of the pandemic and vaccine safety have alarmingly spread among the general population [10]. Therefore, several concerns about the vaccine's safety and side effects have been raised and were the main obstacles associated with unwillingness to accept the COVID-19 vaccine [11].

Although the COVID-19 vaccine represents the primary hope for preventing the spread of the infection, it is important to note that no vaccine is entirely devoid of side effects. Individuals have reported a spectrum of responses to vaccines, ranging from minimal to severe adverse effects. Regardless of any side effects, vaccination provides immunity against COVID-19 disease [12]. The potential side effects following vaccination significantly contribute to the population's vaccine hesitancy. These concerns can be addressed by enhancing public awareness of vaccine safety and efficacy, including the disclosure of potential adverse effects [13,14].

This study sought to assess the factors influencing the severity and occurrence of post-vaccination side effects among various demographic groups in Iraqi Kurdistan. Specifically, it examined the impact of pre-existing health conditions, prior COVID-19 infections, and the type of vaccine administered on the development of side effects. By expanding the analysis to include a wider range of demographic and clinical variables, this research aimed to overcome the limitations of earlier studies and offer targeted public health recommendations to improve vaccine safety and acceptance in the region.

## Materials and methods

Study design and participants

A population-based study took place in Duhok province, Kurdistan Region of Iraq, from September 2022 to April 2023. Data acquisition occurred through two methods: face-to-face interviews conducted by one of the authors in public places, hospitals, and universities or electronically via the Google Forms platform (Google LLC, Mountain View, California, USA).

The study’s sample size was determined using an online calculator (http://www.raosoft.com/samplesize.html) with a confidence interval of 99%, a 5% margin of error, a population size of 1.5 million, and a response distribution of 50%. The calculator recommended a minimum sample size of 664 for the survey. However, the study enrolled a total of 1340 individuals, which represents twice the recommended sample size. The sample size was increased to strengthen the statistical analysis and ensure the reliability of the study’s results.

Study questionnaire

The study questionnaire was designed based on a previously validated questionnaire with slight modifications made by the author team to align with the objectives of the study [15]. This questionnaire encompassed 21 items that were categorized into two distinct sections: The initial section consisted of 10 items designed to collect data regarding the demographical characteristics of the participants. These included age, gender, marital status, occupation, adherence to a healthy lifestyle, smoking habits, any history of chronic illnesses, any type of allergy, blood group, and the sources from which participants obtained information about COVID-19 vaccines.

The second section of the questionnaire consisted of 11 items to gather information about participants' COVID-19 infection and vaccination status. Vaccination status questions included the type of vaccine received, number of doses, development of post-vaccination side effects, duration and severity of the side effects, the specific dose at which side effects occurred, and whether they had contracted COVID-19 after vaccination. Post-vaccination side effects were further categorized into two groups: local and systemic side effects. The local side effects included pain, edema, itching, redness, and hotness at the injection site. Systemic side effects included a list of symptoms such as headache, fatigue, fever, chills, joint pain, muscle pain, diarrhea, nausea, and hair loss. In addition, participants had the opportunity to report any other symptoms not mentioned in the provided list. To assess the severity of side effects, a Likert scale from 1 to 10 was used, with scores of 1 to 3 considered mild, 4 to 7 classified as moderate, and 8 to 10 categorized as severe. The complete study questionnaire is provided in the appendix.

Participant eligibility criteria

The eligibility criteria encompassed individuals aged 18 years or older, living in Duhok province, who had received at least one dose of COVID-19 vaccines distributed by the Iraqi Kurdistan Ministry of Health, and who consented to participate in the study.

Ethical approval

The final study protocol and design received formal approval from the Ethics and Scientific Committee of the College of Medicine, University of Zakho in Kurdistan Region, Iraq on July 5, 2022, with reference number (JUL2022/E04). Written informed consent was obtained from all individuals.

Statistical analysis

GraphPad Prism version 8.0 (GraphPad Software, Boston, Massachusetts USA) was used to perform statistical analysis. Descriptive statistics were calculated as frequencies and percentages. The mean and standard deviation were calculated for the numerical variables. The association between demographic characteristics and the development and severity of post-vaccination side effects was studied by the Chi-square test. Logistic regression analysis was employed to investigate factors associated with the likelihood of developing post-vaccination side effects. A p-value less than 0.05 was considered statistically significant.

## Results

Demographic characteristics and health profile

The study recruited a total of 1340 participants who received COVID-19 vaccine. The mean age of the participants was (29.21 ± 13.09) years old. Females accounted for 52.76% of the study participants, and roughly two-thirds of the study participants were single. Half of the study participants were students, and 57.76% maintained a healthy lifestyle. One-fifth of the participants reported being smokers, and only 15.52% had chronic health problems. Approximately one-fifth of the participants reported having some form of allergy. Blood groups O and A were the most prevalent among the study participants. Table 1 summarizes demographic and health characteristics of the study respondents.

**Table 1:** Demographic characteristics of the study’s participants SD: standard deviation

Variables	Frequency	%
Age (year)		
18-25	811	60.52
26-35	224	16.72
36-50	171	12.76
51-60	77	5.75
>60	57	4.25
Mean ± SD	29.21±13.09	
Gender		
Male	633	47.24
Female	707	52.76
Marital status		
Single	899	67.09
Married	441	32.91
Occupation		
Healthcare worker	197	14.7
Non-healthcare worker	427	31.87
Student	716	53.43
Do you maintain a healthy lifestyle?		
Yes	774	57.76
No	566	42.24
Smoking		
Yes	300	22.39
No	1040	77.61
Chronic health problems		
Yes	208	15.52
No	1132	84.48
Had any type of allergy		
Yes	290	21.64
No	1050	78.36
Blood group		
A	463	34.55
B	257	18.18
O	485	36.19
AB	135	10.07

Source of knowledge about COVID-19 vaccines

**Figure 1:**
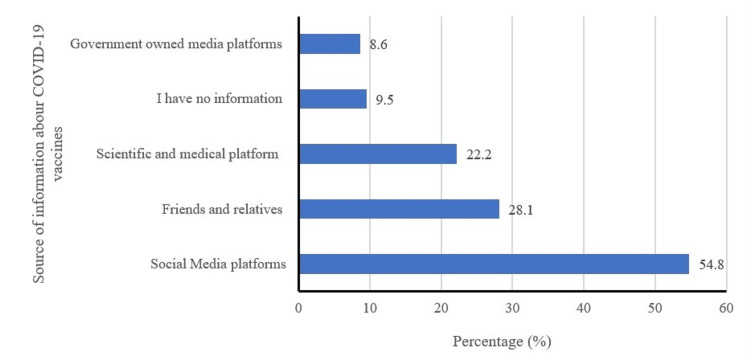
Source of information about COVID-19 vaccines (can choose multiple answers)

Figure 1 illustrates the primary sources of information on COVID-19 vaccines. Interestingly, it was observed that over half of the participants obtained information from social media platforms, while approximately one-third acquired information from friends and relatives. By contrast, only 22.2% accessed information from scientific and medical platforms, and a mere (8.6%) relied on government-owned media platforms.

COVID-19 infection and vaccination data

Table 2 highlights data regarding participants' past COVID-19 infections and vaccination data. Three-fifths of the study participants had experienced a prior COVID-19 infection. Two-thirds of the participants received the Pfizer/BioNTech vaccine, and a significant majority (76.87%) completed the two-dose vaccination regimen. Notably, around one-third (32.4%) reported contracting COVID-19 after vaccination, with a majority indicating that the infection occurred following the second dose of vaccination. The majority of the participants, accounting for 76.94% experienced side effects following administration of the COVID-19 vaccine with the mean duration of the side effects (2.8 ± 1.92 days). Approximately one-third of the participants encountered mild symptoms after vaccination, while 11.12% reported severe symptoms.

**Table 2:** History of COVID-19 infection and vaccination data among vaccinated population SD: standard deviation

COVID-19 infection and vaccination data	Frequency	%
Previously had a positive test for COVID-19		
Yes	816	60.9
No	524	39.1
Type of vaccine		
Pfizer/BioNTech	909	67.84
Oxford/AstraZeneca	261	19.48
Sinopharm	170	12.69
Number of doses of COVID-19 vaccine received		
One dose	181	13.51
Two doses	1030	76.87
Three doses	129	9.63
Infected with COVID-19 after vaccination		
Yes	434	32.39
No	906	67.61
Infected with COVID-19 after which dose		
1st dose	142	32.72
2nd dose	255	58.76
3rd dose	37	8.52
Post-vaccination side effects		
Yes	1031	76.94
No	309	23.06
Duration of symptoms after vaccination (Day Mean±SD)	2.8±1.92	
Severity of side effects after vaccination		
Mild	493	36.79
Moderate	389	29.03
Severe	149	11.12
No symptoms	309	23.06

Comparison of post-vaccination side effects based on the type of vaccine

Table 3 provides a summary of post-vaccination side effects associated with various COVID-19 vaccines. When comparing these side effects based on the vaccine type received, it was observed that pain and hotness at the injection site were the most common local reactions. These were more prevalent after the first dose of the vaccine and in participants who received Oxford/AstraZeneca or Pfizer/BioNTech vaccines compared to participants who received the Sinopharm vaccine. In addition, other local side effects after the first dose were more pronounced in participants who received the Oxford/AstraZeneca vaccine.

**Table 3:** Prevalence of post-vaccination side effects based on the type of vaccine administered

Type of vaccine	Side effects	After the first dose	After the second dose	After both doses	None
		n (%)	n (%)	n (%)	n (%)
Pfizer-BioNTech	Systemic side effects				
(n = 909)	Headache	182 (20.02)	99 (10.89)	48 (5.28)	580 (63.81)
	Fatigue	174 (19.14)	92 (10.12)	53 (5.83)	590 (64.91)
	Fever	251 (27.61)	131 (14.41)	60 (6.61)	467 (51.38)
	Chills and tremors	57 (6.27)	34 (3.74)	16 (1.76)	802 (88.22)
	Joint pain	71 (7.81)	47 (5.17)	35 (3.85)	756 (83.17)
	Myalgia	114 (12.54)	54 (5.94)	33 (3.63)	708 (77.89)
	Diarrhea	16 (1.76)	11 (1.21)	11 (1.21)	871 (95.82)
	Nausea	27 (2.97)	14 (1.54)	13 (1.43)	855 (94.06)
	Local side effect				
	Local pain	267 (29.37)	128 (14.08)	72 (7.92)	442 (48.62)
	Local edema	46 (5.06)	25 (2.75)	14 (1.54)	824 (90.65)
	Itching	21 (2.31)	15 (1.65)	6 (0.66)	867 (95.38)
	Local redness	42 (4.62)	25 (2.75)	9 (0.99)	833 (91.74)
	Local hotness	77 (8.47)	49 (5.39)	25 (2.75)	758 (83.39)
Oxford/AstraZeneca	Systemic side effects				
(n = 261)	Headache	68 (26.05)	68 (26.05)	5 (1.92)	120 (45.98)
	Fatigue	71 (27.20)	19 (7.28)	10 (3.83)	161 (61.69)
	Fever	95 (36.39)	33 (12.64)	16 (6.13)	117 (44.83)
	Chills and tremors	41 (15.71)	8 (3.07)	3 (1.15)	209 (80.08)
	Joint pain	10 (3.83)	19 (7.28)	6 (2.29)	226 (86.59)
	Myalgia	36 (13.79)	20 (7.66)	12 (4.60)	193 (73.95)
	Diarrhea	11 (4.21)	2 (0.77)	0 (0.0)	248 (95.02)
	Nausea	24 (9.20)	6 (2.30)	4 (1.53)	227 (86.97)
	Local side effect				
	Local pain	87 (33.33)	35 (14.41)	21 (8.05)	118 (45.21)
	Local oedema	16 (6.13)	7 (2.68)	6 (2.30)	232 (88.89)
	Itching	17 (6.51)	5 (1.92)	4 (1.53)	235 (90.04)
	Local redness	18 (6.90)	8 (3.07)	4 (1.53)	231 (88.51)
	Local hotness	30 (11.49)	7 (2.68)	4 (1.53)	220 (84.29)
Sinopharm	Systemic side effects				
(n = 170)	Headache	23 (13.53)	13 (7.65)	15 (8.82)	119 (70.0)
	Fatigue	18 (10.59)	14 (8.24)	23 (13.53)	115 (67.65)
	Fever	22 (12.94)	25 (14.71)	17 (10.0)	106 (62.35)
	Chills and tremors	4 (2.35)	4 (2.35)	8 (4.71)	154 (90.59)
	Joint pain	10 (5.88)	12 (7.06)	10 (5.88)	138 (81.18)
	Myalgia	11 (6.47)	9 (5.29)	12 (7.06)	138 (81.18)
	Diarrhea	5 (2.94)	2 (1.18)	2 (1.18)	161 (94.71)
	Nausea	5 (2.94)	6 (3.53)	3 (1.76)	156 (91.76)
	Local side effect				
	Local pain	28 (16.47)	20 (11.76)	18 (10.58)	104 (61.18)
	Local edema	9 (5.29)	5 (2.94)	11 (6.47)	145 (85.29)
	Itching	2 (1.18)	7 (4.12)	3 (1.76)	158 (92.94)
	Local redness	7 (4.12)	3 (1.76)	4 (2.35)	156 (91.76)
	Local hotness	13 (7.65)	5 (2.94)	11 (6.47)	141 (82.94)

The most common systemic side effects included fever, headache, and fatigue. Interestingly, systemic side effects reported after the first dose of Sinopharm were lower than those observed with either Oxford/AstraZeneca or Pfizer/BioNTech vaccines. However, all systemic side effects were more prominent with the Oxford/AstraZeneca vaccine compared to the Pfizer/BioNTech vaccine, except joint pain.

When examining side effects associated with the second dose of COVID-19 vaccines, there was a significant reduction in the incidence of side effects compared to the first dose. Nevertheless, itching at the injection site notably increased with the second dose of Sinopharm. Furthermore, fever after the second dose of Sinopharm was more prevalent than with Oxford/AstraZeneca and Pfizer/BioNTech.

Severity of post-vaccination side effects

Table 4 presents an analysis of the severity of adverse effects based on participants' demographic characteristics, health profiles, and vaccination data. The majority of participants in the age groups of 36-50 and 51-60 years experienced side effects following vaccination (p-value < 0.001). Married participants, by contrast, tended to experience more severe side effects compared to unmarried participants. Occupational differences were also associated with side effects severity (p-value < 0.001), with approximately two-thirds of students reporting either no or mild side effects.

**Table 4:** Severity of post-vaccination side effects according to demographic characteristics, health profile, and vaccination data The p-value was calculated using the chi-square test.

Variables	Mild n (%)	Moderate n (%)	Severe n (%)	No side effect n (%)	p-value
Age (year)					
18-25	318 (39.21)	217 (26.76)	68 (8.38)	208 (25.65)	<0.001
26-35	68 (30.36)	79 (35.27)	32 (14.29)	45 (20.09)	
36-50	63 (36.84)	51 (29.83)	28 (16.37)	29 (16.96)	
51-60	22 (28.57)	30 (38.96)	12 (15.58)	13 (16.88)	
>60	22 (38.60)	12 (21.05)	9 (15.79)	14 (24.56)	
Gender					
Male	233 (36.81)	178 (28.12)	63 (9.95)	159 (25.12)	0.26
Female	260 (36.78)	211 (29.84)	86 (12.16)	150 (21.22)	
Marital status					
Single	335 (37.26)	247 (27.47)	85 (9.45)	232 (25.81)	< 0.001
Married	158 (35.83)	142 (32.20)	64 (14.51)	77 (17.46)	
Occupation					
Healthcare worker	71 (36.04)	67 (34.01)	25 (12.69)	34 (17.26)	<0.001
Non-healthcare worker	143 (33.49)	140 (32.79)	66 (15.46)	78 (18.27)	
Student	279 (38.97)	182 (25.42)	58 (8.10)	197 (27.51)	
Do you maintain a healthy lifestyle?					
Yes	291 (37.60)	226 (29.20)	81 (10.47)	176 (22.74)	0.78
No	202 (35.69)	163 (28.79)	68 (12.01)	133 (23.49)	
Smoking					
Yes	113 (37.67)	87 (29.0)	28 (9.33)	72 (24.0)	0.72
No	380 (36.54)	302 (29.04)	121 (11.63)	237 (22.79)	
Chronic health problems					
Yes	62 (29.81)	76 (36.54)	30 (14.42)	40 (19.23)	0.007
No	431 (38.07)	313 (27.65)	119 (10.51)	269 (23.76)	
Had any type of allergy					
Yes	94 (32.41)	83 (28.62)	42 (14.48)	71 (24.48)	0.07
No	399 (38.0)	306 (29.14)	107 (10.19)	238 (22.67)	
Blood group					
A	197 (42.55)	101 (21.81)	46 (9.94)	119 (25.70)	<0.001
B	88 (34.24)	73 (28.40)	29 (11.28)	67 (26.07)	
O	172 (35.46)	163 (33.61)	55 (11.34)	95 (19.59)	
AB	36 (26.67)	52 (38.51)	19 (14.07)	28 (20.74)	
Infected with COVID-19 before vaccination					
Yes	304 (37.25)	262 (32.11)	106 (12.99)	144 (17.65)	<0.001
No	189 (36.07)	127 (24.24)	43(8.21)	165 (31.49)	
Type of vaccine received					
Pfizer-BioNTech	344 (37.84)	280 (30.80)	83 (9.13)	202 (22.22)	
Oxford/AstraZeneca	78 (29.88)	84 (32.18)	44 (16.86)	55 (21.07)	<0.001
Sinopharm	71 (41.76)	25 (14.71)	22 (12.94)	52 (30.59)	
Infected with COVID-19 after vaccination					
Yes	163 (37.56)	157 (36.18)	55 (12.67)	59 (13.59)	<0.001
No	330 (36.42)	232 (25.61)	94 (10.38)	250 (27.59)	
Total	493 (36.79)	389 (29.33)	149 (10.82)	309 (33.33)	

The severity of side effects was significantly influenced by chronic health problems (p-value = 0.007). Participants with chronic health issues were more likely to experience moderate and severe side effects compared to those without such problems. A noteworthy association was found between the blood group and side effects severity (p-value < 0.001). Individuals with blood group A tended to have less severe symptoms, with 25.7% experiencing no side effects and 42.55% developing mild side effects. Conversely, those with blood group AB had more severe symptoms, with 38.51% and 14.07% experiencing moderate and severe side effects, respectively.

Participants who had previously contracted COVID-19 before vaccination reported more severe symptoms compared to those without prior infection, and this association was statistically significant (p-value < 0.001). The type of vaccine administered also played a crucial role in side effects severity (p-value < 0.001). Individuals who received the Oxford/AstraZeneca vaccine were more prone to developing moderate to severe side effects compared to their counterparts. Conversely, over two-thirds of those who received the Sinopharm vaccine reported either no side effects or mild ones. Notably, no significant associations were found between the severity of post-vaccination side effects and gender, maintenance of a healthy lifestyle, smoking status, or any type of allergy.

**Figure 2:**
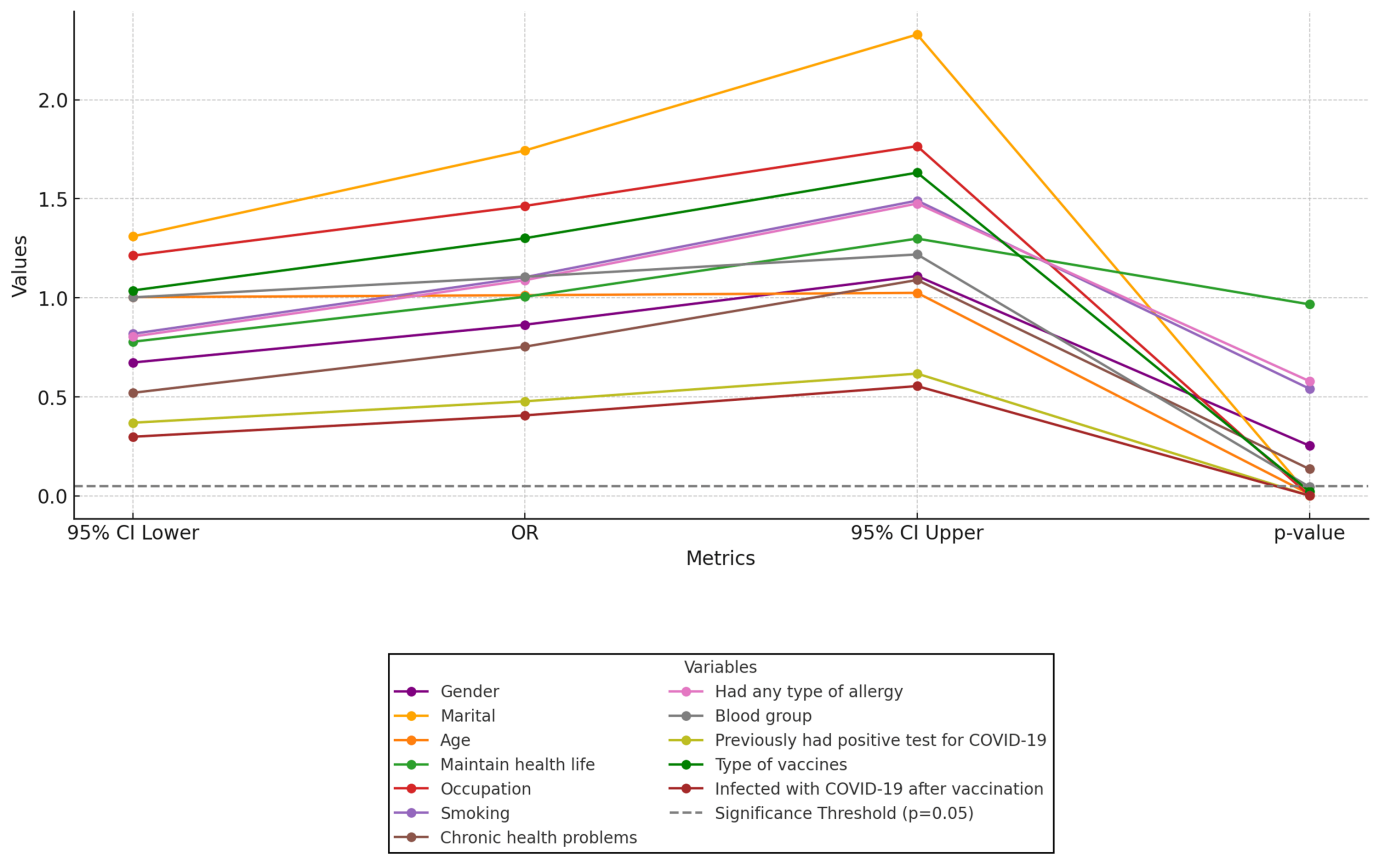
Parallel coordinate plot of logistic regression for post-vaccination side effects

Figure 2 presents the results of multiple logistic analyses for the study variables, highlighting significant factors associated with post-vaccination side effects. Marital status, age, occupation (particularly healthcare workers), blood group O, a previous positive COVID-19 test, and receiving the Oxford/AstraZeneca vaccine were all linked to an increased likelihood of experiencing side effects. Conversely, gender, maintaining a healthy lifestyle, smoking, chronic health conditions, and allergies did not show a significant association with the likelihood of side effects.

Incidence of infection before and after COVID-19 vaccine 

**Figure 3:**
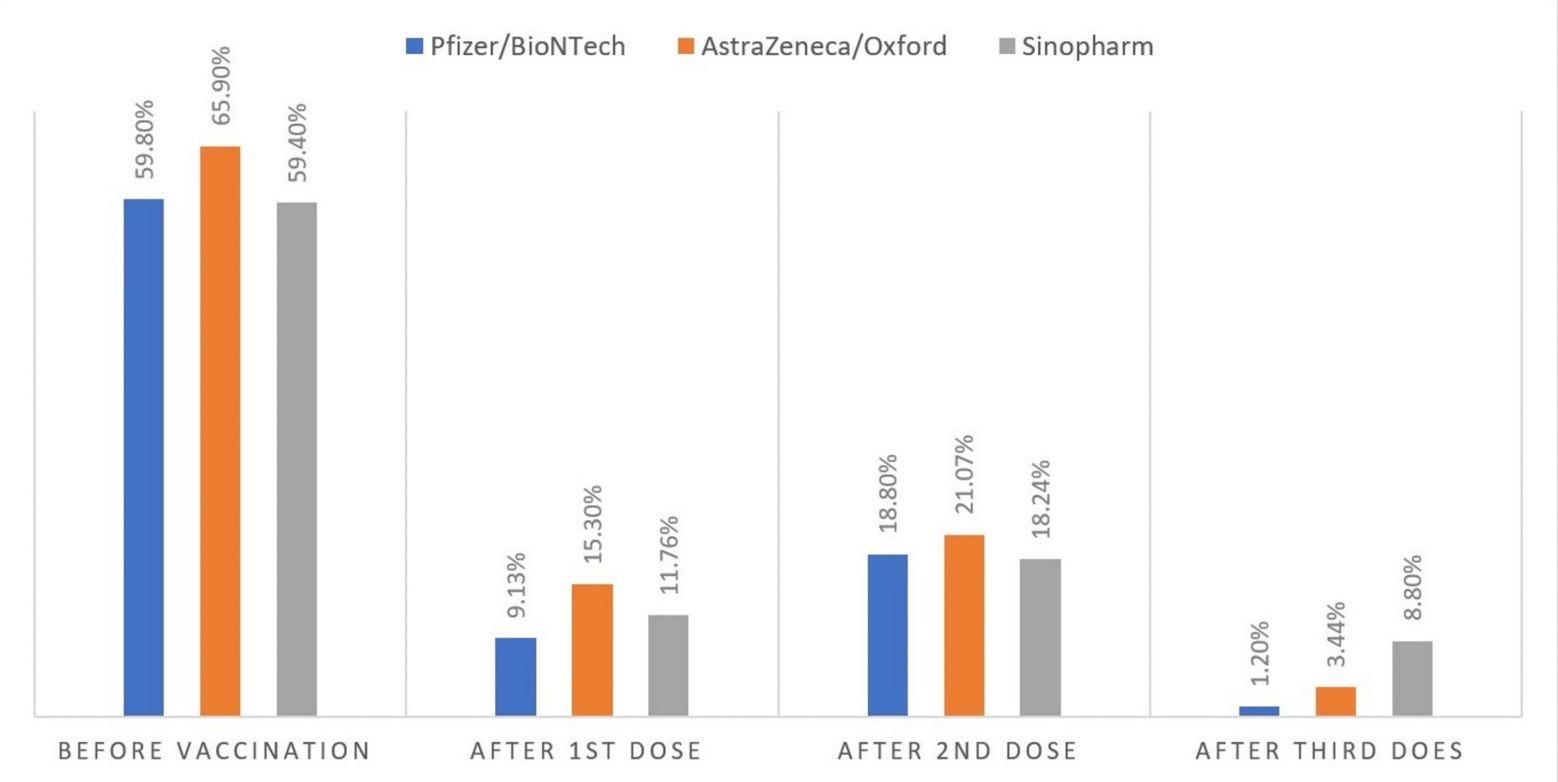
COVID-19 infection before and after vaccination

Figure 3 illustrates the prevalence of COVID-19 infection among the study participants before and after vaccination. Approximately three-fifths of the total participants who received the Pfizer/BioNTech vaccine had encountered COVID-19 infection before vaccination. Following the administration of the Pfizer/BioNTech vaccine, there was a marked reduction in the incidence of COVID-19 infection, with only 9.13% and 18.8% developing infection after the first and second doses of the vaccine, respectively. Importantly, the incidence of infection significantly dropped to just 1.2% after the third (booster) dose.

For individuals who received the Oxford/AstraZeneca vaccine, two-thirds had experienced COVID-19 infection before vaccination. The administration of the following vaccine led to a reduction in the incidence of COVID-19 infection to 15.3%, 21.7%, and 3.44% after the first, second, and third (booster) doses, respectively. The Sinopharm vaccine also contributed to a decline in the rate of COVID-19 infection, although the highest incidence after the third (booster) dose was reported among individuals who received this vaccine. Notably, recipients of the Pfizer/BioNTech vaccine exhibited a lower incidence of COVID-19 infection after vaccination compared to those who received the Oxford/AstraZeneca or Sinopharm vaccines.

## Discussion

Throughout history, vaccination has played an essential role in the prevention and eradication of infectious diseases. In the face of the COVID-19 pandemic, vaccination emerged as a pivotal strategy to curb the spread of the disease, alleviate its impact on public health, and mitigate economic crises. The development of effective vaccines has emerged as a global health priority to mitigate infection rates, mortality, and hospitalizations [16,17]. Studies have demonstrated that vaccinated patients exhibit a lower likelihood of severe chest involvement, oxygen requirement, and mortality compared to their unvaccinated counterparts. In a remarkably short timeframe, several vaccines from multiple different companies have been available worldwide [16,17]. However, vaccines were not free from side effects, and at this point, several rumors regarding vaccine safety were circulating in the communities. These misconceptions acted as a hindrance to achieving high vaccination rates. Understanding the safety and side effects of COVID-19 vaccines is essential worldwide to address public concerns and ensure broad vaccine acceptance. This study aimed to identify potential side effects of COVID-19 vaccines, highlighting the global need for ongoing monitoring and analysis of vaccine safety and efficacy in every country.

In the present study, around half of the participants indicated that their information on COVID-19 vaccines came from social media platforms. Interestingly, this contrasts with a study conducted in Saudi Arabia where half of the participants relied on government-owned media platforms for COVID-19 vaccine information [18]. The study found a relatively high prevalence of COVID-19 infection among participants, standing at 60.9%. This contrasts with previous studies conducted in the Kurdistan Region and Iraq, which reported a lower percentage of individuals with a positive history of COVID-19 infection [19,20]. The high prevalence observed in our study could be attributed to its recency compared to other studies, and potentially being influenced by the emergence of more COVID-19 variants and waves in the region.

In our study, approximately three-fourths of the participants experienced post-vaccination side effects, showing slightly higher rates compared to another study conducted in Iraq [19]. Among those who developed side effects after vaccination, 37% reported mild side effects, while 29% and 11% reported moderate and severe side effects, respectively. This aligns with a study conducted in Saudi Arabia, where mild to moderate side effects were commonly reported after COVID-19 vaccination [18]. Severe side effects were observed in around one-tenth of our study participants, consistent with previous studies indicating that approximately one-tenth of vaccinated individuals may experience severe side effects [18].

The most frequently reported side effects by participants in the current study included injection site pain, fever, headache, and fatigue. These findings align with studies conducted in neighboring countries [21,22]. In our study, individuals who received the Oxford/AstraZeneca vaccine experienced injection site pain more frequently, followed by Pfizer/BioNTech and Sinopharm. This difference arises from the distinct mechanisms of the vaccines; AstraZeneca’s viral vector platform uses adenovirus to deliver the spike protein, often provoking a stronger immune response and more noticeable side effects than mRNA vaccines like Pfizer /BioNTech [23]. However, a study in Bahrain found that pain at injection sites is more commonly observed after the Pfizer/BioNTech vaccine compared to AstraZeneca/Oxford and Sinopharm [24]. An Egyptian study indicated that side effects were more frequently observed after the first dose of vaccination with Pfizer/BioNTech, AstraZeneca/Oxford, and Sinopharm compared to the second dose, a pattern similar to what we observed in our study [25]. Furthermore, individuals who received the Pfizer-BioNTech vaccine in our study reported more side effects compared to those who received other vaccines. These findings are consistent with studies conducted in Iran and Syria [21,26].

Although a statistically significant association was not identified, the percentage of females experiencing side effects was higher than that of males, aligning with similar findings reported in a study from Saudi Arabia [27]. Moreover, a study conducted in Iran found that post-vaccination side effects were more frequently observed in females following the administration of the Sinopharm vaccine [22]. Participants with chronic health conditions were also more prone to experiencing moderate to severe side effects after vaccination, a result consistent with studies conducted in Syria and the UAE [15,21]. However, a study in Iraq reported that side effects were more pronounced among individuals without comorbidities [20]. Having a history of COVID-19 infection before vaccination was identified as another determinant of developing side effects after COVID-19 vaccination, aligning with findings reported in studies from Iraq and Turkey [19,28]. Nonetheless, a study in Saudi Arabia found that side effects were more pronounced in individuals with no previous history of infection [27].

All three vaccine types administered to the study participants have contributed to a reduction in COVID-19 infections. Following the second dose of the Pfizer/BioNTech, AstraZeneca/Oxford, and Sinopharm vaccines, the incidence of infection decreased to 18.8%, 21.07%, and 18.24%, respectively. However, a study in Iraq reported a lower incidence of infection after the second dose of Pfizer/BioNTech, AstraZeneca/Oxford, and Sinopharm to 5.6%, 9.1%, and 13.6%, respectively [29]. The variation in the incidence of COVID-19 infection after vaccination between our studies may be attributed to the timing of this study, conducted after the emergence of the Omicron variant, against which vaccines were found to be less effective, as a study from Iraqi Kurdistan has documented an outbreak of the Omicron variant among vaccinated healthcare workers [30].

Limitations

The study gained strength from its large sample size, which effectively assessed adverse effects linked to the COVID-19 vaccine within the Iraqi Kurdish population. It stands out as one of the limited studies investigating post-vaccination side effects in the Kurdistan Region of Iraq. Notwithstanding these strengths, certain limitations are worth considering. First, the study's confinement to a single province in Iraqi Kurdistan limits the generalizability of the findings to a national level. Second, relying on self-reported side effects introduces the potential for reporting bias and recall bias, as the participants may not accurately recall all the symptoms they experienced or the duration, acknowledging a need for caution in interpreting the results. Lastly, the study predominantly concentrated on short-term side effects. To comprehensively explore long-term adverse effects associated with COVID-19 vaccines, we propose the undertaking of a nationwide cohort study.

## Conclusions

The study found that fever, headache, and fatigue were the most commonly reported side effects of all three types of vaccines: Pfizer/BioNTech, Oxford/AstraZeneca, and Sinopharm. Encouragingly, three-fifths of the participants experienced either no side effects or mild ones, suggesting a positive trend in promoting COVID-19 vaccine acceptance within our community. 

Notably, side effects were more frequently documented after the initial vaccine dose compared to the second. Among the vaccine recipients, Pfizer-BioNTech vaccine recipients were more prone to experiencing side effects, followed by Oxford/AstraZeneca and Sinopharm recipients. Determinants for experiencing side effects after vaccination included participants aged 36-60, females, individuals with chronic health problems, and those previously infected with COVID-19.

## Appendices

Survey on the side effects of COVID-19 vaccines

By signing below, I hereby confirm my enrollment in this study

Signature _________________

Part 1. Sociodemographic Characteristics

Variables

Age (year)

Gender

Male

Female

Marital status

Single

Married

Occupation

Healthcare worker

Non-healthcare worker

Student

Do you maintain a healthy lifestyle?

Yes

No

Smoking

Yes

No

Chronic health problems

Yes

No

Had any type of allergy

Yes

No

Blood group

A

B

O

AB

Part 2. Source of Information About COVID-19 Vaccination

Social media platforms

Government-owned media platforms

Scientific and medical platform 

Friends and relatives

I have no information

Part 3. COVID-19 Infection and Vaccination Data

Previously had a positive test for COVID-19

Yes

No

Type of vaccine

Pfizer/BioNTech

Oxford/AstraZeneca

Sinopharm

Number of doses of COVID-19 vaccine received

One dose

Two doses

Three doses

Infected with COVID-19 after vaccination

Yes

No

Infected with COVID-19 after which dose 

Not Infected

1st dose

2nd dose

3rd dose

Post-vaccination side effects

Yes

No

Side effects occurred after which dose

No side effects

1st dose

2nd dose

3rd dose

Local side effects

Local pain

Local oedema

Itching

Local redness

Local hotness

Systemic side effects

Headache

Fatigue

Fever

Chills

Joint pain

Myalgia

Diarrhea

Nausea

Duration of symptoms after vaccination in days

Severity of side effects after vaccination (use the scale from 0 to 10) 
